# 4047 EEG as a Predictor of Post-Stroke Recovery: A Systematic Review and Meta-Analysis

**DOI:** 10.1017/cts.2020.233

**Published:** 2020-07-29

**Authors:** Amanda Vatinno, Viswanathan Ramakrishnan, Annie Simpson, Heather Bonilha, Na Jin Seo

**Affiliations:** 1 Medical University of South Carolina

## Abstract

OBJECTIVES/GOALS: The objective of this study is to perform a systematic review and meta-analysis on the prognostic utility of electroencephalography (EEG) in stroke recovery. METHODS/STUDY POPULATION: A literature search was conducted using three electronic databases, including PubMed, Scopus, and CINAHL. Key search terms were “EEG,” “stroke,” and “rehabilitation”. Only peer-reviewed journal articles published in English that examined the relationship between EEG and a standardized clinical outcome measure(s) at a later time in stroke patients were included. Two independent raters completed data extraction and assessed methodological quality of the studies with the Downs and Black form. A linear meta-regression was performed across subsets of individual studies that utilized a common clinical outcome measure to determine the association between EEG and clinical outcome while adjusting for sample size and study quality. RESULTS/ANTICIPATED RESULTS: 56 papers met the inclusion criteria and were included in the systematic review. The prognostic value of EEG was evidenced at both the acute and chronic stages of stroke. The addition of EEG enhanced prognostic accuracy more than initial clinical assessment scores and/or lesion volume alone. In the meta-analysis, a subset of 10 papers that utilized the National Institutes of Health Stroke Scale (NIHSS) and a subset of 7 papers that utilized the Modified Rankin Scale (MRS) were included. Analysis demonstrated an association between EEG and the subsequent clinical outcome measures. DISCUSSION/SIGNIFICANCE OF IMPACT: Currently, prognosis is largely based on initial behavioral impairment level. However, post-stroke recovery outcomes are heterogeneous despite similar initial clinical presentations. Uncertain prognosis makes it difficult for clinicians to develop personalized treatment plans for patients. Improved prognosis for recovery may guide clinical management for stroke survivors by helping clinicians determine the maximally efficient course of treatment and care. This study suggests that prognostic accuracy may be enhanced using EEG.

